# Generalisability and potential deaths averted from intensive blood pressure treatment among the elderly population in the US and China: A nationally representative cross-sectional study

**DOI:** 10.7189/jogh.13.04100

**Published:** 2023-09-08

**Authors:** Chao Li, Chenyu Yang, Fang Shao, Xuanyi Liang, Kangyu Chen, Tian Yang, Zhenqiang Wu, Guoshuai Shi, Tao Chen

**Affiliations:** 1 Department of Epidemiology and Health Statistics, School of Public Health, Xi’an Jiaotong University Health Science Center, Xi’an, China; 2 School of Public Health, Xi’an Medical College, Xi'an, China; 3 Department of Biostatistics, School of Public Health, Nanjing Medical University, Nanjing, China; 4 Department of Cardiology, The First Affiliated Hospital of USTC, Division of Life Sciences and Medicine, University of Science and Technology of China, Hefei, China; 5 Department of Respiratory and Critical Care Medicine, The First Affiliated Hospital of Xi’an Jiaotong University, Xi’an, China; 6 Department of Geriatric Medicine, The University of Auckland, Auckland, New Zealand; 7 Centre for Health Economics, University of York, Heslington, United Kingdom

## Abstract

**Background:**

The Systolic Blood Pressure Intervention Trial (SPRINT) from the US and the Strategy of Blood Pressure Intervention in the Elderly Hypertensive Patients (STEP) trial from China have consistently demonstrated clinical benefits from intensive blood pressure (BP) treatment among elderly adults with hypertension. However, we have little data on the generalisability and potential implications of a scale-up of intensive BP treatment to all eligible elderly in the US and China.

**Methods:**

We used two nationally representative data sets from China (Health and Retirement Longitudinal Study (CHALRS), 2011-2012) and the US (National Health and Nutrition Examination Survey (NHANES), 2007-2012) and linked them with CHARLS follow-up data (2013) and the National Death Index (1999-2015), respectively. We estimated the percentage, number, and characteristics of elderly (≥60 years old) meeting the STEP and SPRINT eligibility criteria, and deaths that would be prevented or postponed with the implementation of intensive BP treatment.

**Results:**

Among the Chinese adults aged 60 years and over, 38.89% (95% confidence interval (CI) = 36.97-40.84) or 85.39 (95% CI = 81.14-89.64) million subjects met the STEP criteria, and 40.90 million (47.90%) adults were not taking antihypertensive medications. In the US, 23.77% (95% CI = 22.32%-25.28) or 12.46 (95% CI = 11.68-13.24) million elderly were eligible for the SPRINT, and 5.78 million (46.36%) were untreated. Overall, 0.07 (95% CI = 0.06-0.08) million deaths in the US and 0.31 (95% CI = 0.25-0.39) in China would be averted annually if intensive BP treatment was implemented, while 120 000 and 680 000 of hypotension cases would be identified yearly in

the US and China, respectively.

**Conclusions:**

A substantial percentage of Chinese and the US elderly meet the eligibility criteria for STEP and SPRINT. If intensive BP treatment was adopted, 70 000 and 310 000 deaths would be prevented or postponed yearly in the US and China, respectively.

Hypertension is the leading risk factor for cardiovascular disease mortality and is exceedingly common in the elderly [1,2]. Significant cardiovascular benefits were reported with intensive blood pressure (BP) control among older patients [3,4]. Two recent large-scale randomised clinical trials from the Strategy of Blood Pressure Intervention in the Elderly Hypertensive Patients (STEP) and Systolic Blood Pressure Intervention Trial (SPRINT) further confirmed that a more rigid or aggressive BP strategy could significantly reduce the rates of fatal and nonfatal major cardiovascular (CVD) events in older adults [5-7].

Recently, we performed an analysis of six randomised clinical trials (including STEP and SPRINT) consisting of 27 414 individuals with hypertension and found that intensive BP treatment reduced major adverse cardiovascular events (MACE) [8]. This was in line with previous meta-analyses reporting CVD benefits from intensive BP treatment [9-11]. Due to emerging evidence, researchers have suggested that BP management be extended to patients, despite the increasing risk for adverse effects, such as hypotension and syncope [12-14]. In fact, the SPRINT trial influenced the development of several international or regional guidelines [15,16]. For example, the 2017 American College of Cardiology/American Heart Association BP guideline recommends a BP target of <130/80 mm Hg for most adults aged ≥65 years.

The generalisability and potential implications of a population-level scale-up of intensive BP treatment to eligible elderly is unclear. Based on data from two nationally representative studies, we aimed to estimate the prevalence and number of elderly patients who met the STEP and SPRINT eligibility criteria, and deaths prevented or postponed per year with the implementation of intensive BP treatment in China and the US.

## METHODS

### Data sources and study population

We used two large, nationally representative data sets from China (the China Health and Retirement Longitudinal Study (CHARLS) [17]) and the US (National Health and Nutrition Examination Survey (NHANES) [18]).

For China, we used the CHARLS 2011-2012 baseline survey data to estimate the percentage and number of elderly patients who met the STEP eligibility criteria. For the US, we pooled data from the 2007-2008, 2009-2010, and 2011-2012 NHANES cycles to provide stable estimates in population subgroups of SPRINT eligibility and ensure comparability with CHARLS data from China. We also linked the 2013 follow-up data in the CHARLS [17] and the National Death Index collected by 31 December 2015 in the NHANES [19] to estimate the number of prevented or postponed deaths.

The details of the CHARLS and NHANES have been described elsewhere [17,18]. Briefly, the CHARLS is an ongoing nationally representative survey in China which is longitudinally following-up people aged >45 years. Interview status (dead or alive) and death date were recorded in the 2013 follow-up [20]. The NHANES is a nationally representative cross-sectional survey of the non-institutionalised US population with data collected in two-year cycles [18]. The CHARLS and NHANES both collected information on demographic characteristics, medical history, prescription drug use, and laboratory testing. Results from the CHARLS and NHANES can be weighted to obtain nationally representative estimates [17,18]. The CHARLS [21] and NHANES [22] data are available their respective websites All participants provided written informed consent.

In this analysis, we excluded missing values of systolic blood pressure (SBP) and diastolic blood pressure (DBP), self-reported history of antihypertensive treatment, participants aged <60 years, and those with uncompleted medical evaluations, leaving 5912 participants in CHARLS and 5508 in NHANES. This analysis was approved by Xi’an Jiaotong University Health Science Centre.

### Study variables

Seated BP for each participant was taken three times at 45-second intervals using Omron digital devices (Omron model HEM-7200) in the CHARLS [17]. Similarly, seated BP was taken thrice at one-minute intervals after five minutes of rest, but was measured by a mercury sphygmomanometer in the NHANES [18].

We defined treated hypertension as participants taking medicines for the management of hypertension and untreated hypertension if they were not taking medicines, but had hypertension.

### STEP and SPRINT eligibility

We used the following SPRINT eligibility criteria [7] to determine the eligibility of elderly for intensive BP treatment in the US:

1. A SBP of 130-180 mm Hg, depending on the number of antihypertensive medication classes being taken (SBP 130-180 mm Hg on none or one antihypertensive medication class, 130-170 mm Hg on up to two classes, 130-160 mm Hg on up to three classes, and 130-150 mm Hg on up to four classes);
2. One or more of the following high CVD risk criteria:
  - Clinical coronary heart disease;
  - Estimated glomerular filtration rate (eGFR) of 20-59 mL/min/1.73 m^2^;
  - Framingham risk score for 10-year CVD risk ≥15%
  - Age ≥75 years.

We excluded patients with diabetes, history of stroke, proteinuria >1 g in 24 hours, heart failure, eGFR<20 mL/min/1.73 m^2^.

**Figure 1:**
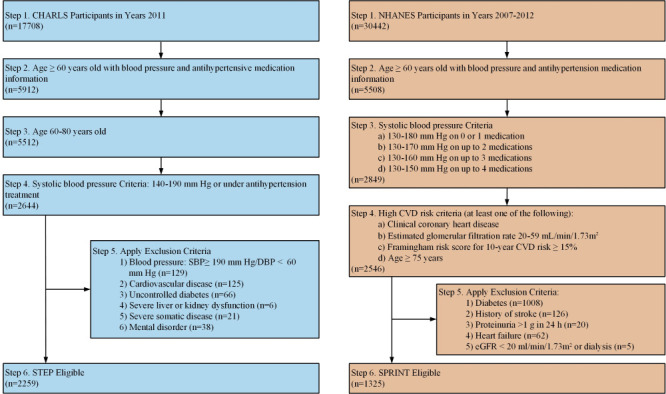
Flowcharts showing the eligibility criteria for STEP and SPRINT. CHARLS – China Health and Retirement Longitudinal Study, DBP – diastolic blood pressure, STEP – Strategy of Blood Pressure Intervention in the Elderly Hypertensive Patients, CVD – cardiovascular disease, eGFR – estimated glomerular filtration rate, NHANES – National Health and Nutrition Examination Survey, SBP – systolic blood pressure, SPRINT – Systolic Blood Pressure Intervention Trial.

Alternatively, we used STEP criteria [6] to determine the eligibility of elderly for intensive BP treatment in China: an age of 60-80 years old, and SBP of 140-190 mm Hg or under antihypertension treatment. We excluded patients with a SBP≥190 mm Hg or DBP<60 mm Hg, those with cardiovascular diseases, uncontrolled diabetes, severe liver or kidney dysfunction, severe somatic disease, or mental disorder (**Figure 1** and Table S1 in the **Online Supplementary Document**).

### Statistical analysis

We estimated the percentage and absolute number of US and Chinese elderly who met the SPRINT and STEP eligibility criteria using sampling weights from the NHANES and CHARLS, extrapolated to US and Chinese populations, correspondingly. We repeated the analysis by sex, age, and BP groups. Furthermore, we calculated the percentage and number of adults with and without treated hypertension among the US and Chinese elderly who met the SPRINT and STEP eligible criteria.

We derived incidence rates of all-cause mortality (per 100 patient-years) for STEP and SPRINT eligible elderly from CHARLS and NHANES, respectively, which we used to calculate the potential deaths averted by intensive BP treatment annually among elderly populations in the US and China after multiplying with pooled hazard ratio (HR) on all-cause mortality from the two trials. We estimated the increased number of hypotension cases from intensive BP treatment in the two countries from the incidence rate difference of hypotension between intensive and standard BP treatment reported in the STEP and SPRINT trials, multiplied with the numbers of SPRINT- and STEP-eligible elderly. All reported *P* values are two-sided with α = 0.05, and all data analyses were conducted using Stata, version 16.0 (StataCorp LLC, College Station, Texas, USA).

## RESULTS

Overall, 38.89% (95% confidence interval (CI) = 36.97-40.84) of Chinese elderly, meaning 85.39 (95% CI = 81.14-89.64) million individuals, met the STEP criteria (**Table 1**). In contrast, there were 28.43% (95% CI = 26.84-30.08) or 14.90 (95% CI = 14.06-15.75) million STEP eligible elderly in the US (Figure S1 in the **Online Supplementary Document**). Among the US elderly, 23.77% (95% CI = 22.32-25.28) or 12.46 (95% CI = 11.68-13.24) million individuals were considered candidates for intensive BP treatment (using the SPRINT criteria) (**Table 2**). In China, 36.26% (95% CI = 34.33-38.24) or 79.62 (95% CI = 75.32-83.91) million elderly were eligible for SPRINT (Figure S1 in the **Online Supplementary Document**).

**Table 1:** Number of China elderly (age ≥60 years) meeting each sequential STEP eligibility criterion*

	Elderly population	Age criteria†	SBP criteria‡	No exclusion criteria§
**Overall population**				
Overall	219.56	200.09 (197.73-202.45)	100.35 (96.07-104.63)	85.39 (81.14-89.64)
Age group in years				
*60-69*	130.25	130.25	60.02 (56.76-63.27)	52.43 (49.09-55.76)
*70-80*	69.84	69.84	40.33 (37.86-42.80)	32.96 (30.50-35.42)
Gender				
*Male*	109.87	102.50 (101.11-103.88)	48.11 (44.78-51.44)	40.64 (37.30-43.98)
*Female*	109.69	97.59 (95.71-99.47)	52.24 (49.51-54.97)	44.75 (42.08-47.41)
SBP, mm Hg				
*140-149*	32.19	29.51 (28.79-30.24)	29.51 (28.79-30.24)	26.33 (25.31-27.35)
*≥150*	58.78	50.25 (48.64-51.86)	48.34 (46.63-50.06)	41.62 (39.41-43.82)
**Treated hypertension**				
Overall	61.01	55.14 (53.75-56.53)	55.14 (53.75-56.53)	44.49 (42.34-46.64)
Age group in years				
*60-69*	34.77	34.77	34.77	28.86 (27.72-30.01)
*70-80*	20.37	20.37	20.37	15.63 (14.34-16.92)
Gender				
*Male*	28.35	26.13 (25.29-26.97)	26.13 (25.29-26.97)	20.81 (19.11-22.51)
*Female*	32.66	29.01 (27.92-30.10)	29.01 (27.92-30.10)	23.68 (22.37-25.00)
SBP, mm Hg				
*140-149*	11.25	10.59 (10.20-10.97)	10.59 (10.20-10.97)	8.82 (8.11-9.53)
*≥150*	25.45	22.06 (20.97-23.15)	22.06 (20.97-23.15)	18.23 (16.76-19.70)
**Without treated hypertension**				
Overall	158.55	144.95 (143.05-146.86)	45.21 (42.21-48.21)	40.90 (38.09-43.70)
Age group in years				
*60-69*	95.48	95.48	25.25 (23.23-27.26)	23.56 (21.58-25.54)
*70-80*	49.47	49.47	19.96 (17.87-22.05)	17.33 (15.40-19.27)
Gender				
*Male*	81.52	76.37 (72.26-77.48)	21.98 (19.83-24.13)	19.83 (17.83-21.84)
*Female*	77.03	68.58 (67.05-70.12)	23.23 (21.13-25.33)	21.06 (19.10-23.02)
SBP, mm Hg				
*140-149*	20.94	18.93 (18.31-19.54)	18.93 (18.31-19.54)	17.51 (16.77-18.25)
*≥150*	33.33	28.19 (27.02-29.36)	26.28 (25.01-27.56)	23.38 (21.75-25.02)

STEP – Strategy of Blood Pressure Intervention in the Elderly Hypertensive Patients, CVD – cardiovascular disease, SBP – systolic blood pressure

*Values presented as millions (95% CI) unless otherwise specified.

†Age 60-80 years old.

‡SBP 140-190 mm Hg or under antihypertension treatment.

§Exclusion criteria: SBP≥190 mm Hg or DBP<60 mm Hg, cardiovascular diseases, uncontrolled diabetes, severe liver or kidney dysfunction, severe somatic disease, mental disorder.

**Table 2:** Number of US elderly (age ≥60 years) meeting each sequential SPRINT eligibility criterion*

	Elderly population	SBP criteria†	High CVD risk‡	No exclusion criteria§
**Overall population**				
Overall	52.41	25.98 (25.04-26.91)	22.30 (21.39-23.21)	12.46 (11.68-13.24)
Age group in years				
*75-79*	6.57	3.67 (3.39-3.94)	3.67 (3.39-3.94)	2.00 (1.74-2.25)
*≥80*	9.42	5.82 (5.51-6.12)	5.82 (5.51-6.12)	3.19 (2.88-3.50)
Gender				
*Male*	23.46	10.91 (10.30-11.52)	10.57 (9.96-11.18)	6.06 (5.53-6.59)
*Female*	28.96	15.06 (14.36-15.77)	11.73 (11.05-12.40)	6.40 (5.83-6.97)
SBP, mm Hg				
*130-139*	10.73	10.73	8.26 (7.86-8.66)	4.49 (4.08-4.89)
*140-149*	7.51	7.51	6.54 (6.27-6.81)	3.75 (3.40-4.10)
*≥150*	8.87	7.74 (7.53-7.94)	7.50 (7.27-7.73)	4.22 (3.87-4.57)
**Treated hypertension**				
Overall	27.75	14.82 (14.17-15.48)	13.66 (13.00-14.31)	6.68 (6.12-7.25)
Age group in years				
*75-79*	4.03	2.43 (2.22-2.64)	2.43 (2.22-2.64)	1.15 (0.95-1.35)
*≥80*	5.94	3.77 (3.53-4.02)	3.77 (3.53-4.02)	1.92 (1.68-2.17)
Gender				
*Male*	11.74	5.81 (5.39-6.24)	5.80 (5.37-6.22)	2.84 (2.47-3.20)
*Female*	16.01	9.01 (8.51-9.50)	7.86 (7.36-8.36)	3.85 (3.41-4.28)
SBP, mm Hg				
*130-139*	5.69	5.69	4.94 (4.72-5.16)	2.36 (2.08-2.64)
*140-149*	4.28	4.28	3.96 (3.78-4.13)	2.11 (1.84-2.37)
*≥150*	5.58	4.85 (4.69-5.01)	4.76 (4.58-4.94)	2.22 (1.94-2.49)
**Without treated hypertension**				
Overall	24.67	11.16 (10.49-11.82)	8.64 (8.03-9.26)	5.78 (5.24-6.31)
Age group in years				
*75-79*	2.53	1.24 (1.06-1.41)	1.24 (1.06-1.41)	0.85 (0.69-1.01)
*≥80*	3.48	2.04 (1.86-2.23)	2.04 (1.86-2.23)	1.27 (1.09-1.45)
Gender				
*Male*	11.72	5.10 (4.66-5.54)	4.77 (4.34-5.21)	3.22 (2.84-3.61)
*Female*	12.95	6.06 (5.56-6.55)	3.87 (3.44-4.30)	2.56 (2.19-2.92)
SBP, mm Hg				
*130-139*	5.04	5.04	3.32 (3.01-3.63)	2.13 (1.83-2.42)
*140-149*	3.23	3.23	2.58 (2.38-2.78)	1.64 (1.41-1.88)
*≥150*	3.30	2.88 (2.75-3.01)	2.74 (2.59-2.89)	2.01 (1.81-2.21)

SPRINT – Systolic Blood Pressure Intervention Trial, CVD – cardiovascular disease, SBP – systolic blood pressure

* Values presented as millions (95% CI) unless otherwise specified.

†SBP 130-180 mm Hg depending on the number of antihypertensive medication classes being taken (SBP 130-180 mm Hg on none or one antihypertensive medication class, 130-170 mm Hg on up to two classes, 130-160 mm Hg on up to three classes, and 130-150 mm Hg on up to four classes).

‡Clinical coronary heart disease, estimated glomerular filtration rate (eGFR) 20-59 mL/min/1.73 m^2^, Framingham risk score for 10-year CVD risk ≥15%, age ≥75 years.

§Exclusion criteria: Diabetes, history of stroke, proteinuria >1g in 24 h, heart failure, or eGFR<20 mL/min/1.73 m^2^.

**Figure 2:**
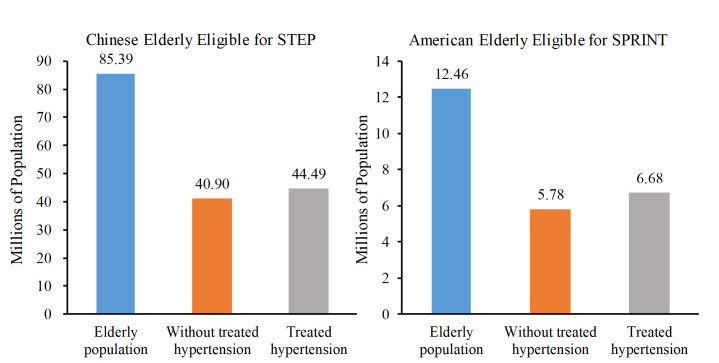
Number of Chinese adults meeting STEP eligibility criteria and US adults meeting SPRINT eligibility criteria. STEP – Strategy of Blood Pressure Intervention in the Elderly Hypertensive Patients, SPRINT – Systolic Blood Pressure Intervention Trial.

Additionally, the elderly in China eligible for STEP tended to be older, female, and to have an SBP≥140 mm Hg (Tables S2 and S3 in the **Online Supplementary Document**). Similarly, SPRINT-eligible elderly in the US tended to be older, male, and to have higher SBP (Tables S2 and S4 in the **Online Supplementary Document**). Notably, 5.78 million (46.36%) of the SPRINT-eligible elderly were not being treated for hypertension. By comparison, 40.90 million (47.90%) STEP-eligible elderly in China were not taking antihypertensive medications (**Figure 2**).

The incidence rate of all-cause death among SPRINT- and STEP-eligible elderly was 2.98 (95% CI = 2.62-3.40) and 1.92 (95% CI = 1.55-2.38), respectively (**Table 3**). The pooled HR of intensive BP treatment (STEP and SPRINT) was 0.81 (95% CI = 0.68-0.97) (Figure S2 in the **Online Supplementary Document**). Thus, we estimated that 0.07 (95% CI = 0.06-0.08) million deaths in the US and 0.31 (95% CI = 0.25-0.39) in China could be averted with the implementation of intensive BP treatment (**Table 3**), although 120 000 and 680 000 of hypotension cases would be identified in the US and China, respectively (Figure S3 in the **Online Supplementary Document**). Additionally, the number of deaths prevented with the implementation of intensive BP treatment among US elderly with treated hypertension was 0.04 (95% CI = 0.03-0.04) compared with 0.03 (95% CI = 0.03-0.04) million without treated hypertension. Correspondingly, there were 0.18 (95% CI = 0.14-0.24) million with treated hypertension and 0.13 (95% CI = 0.10-0.18) million without treated hypertension in China (Table S5 in the **Online Supplementary Document**).

**Table 3:** Potential deaths averted from intensive blood pressure treatment among elderly population in the China and the US*

	Populations	Death rate per 100 person-years	Standard group	Intensive BP group	Difference
**China**					
Overall	85.39	1.92 (1.55-2.38)	1.64 (1.33-2.03)	1.33 (1.08-1.65)	0.31 (0.25-0.39)
Age group in years					
*75-79*	52.43	1.26 (0.91-1.74)	0.66 (0.48-0.91)	0.53 (0.39-0.74)	0.13 (0.09-0.17)
*≥80*	32.96	3.14 (2.38-4.16)	1.04 (0.78-1.37)	0.84 (0.63-1.11)	0.20 (0.15-0.26)
Gender					
*Male*	40.64	2.44 (1.85-3.22)	0.99 (0.75-1.31)	0.80 (0.61-1.06)	0.19 (0.14-0.25)
*Female*	44.75	1.47 (1.06-2.05)	0.66 (0.47-0.92)	0.53 (0.38-0.74)	0.13 (0.09-0.17)
SBP, mm Hg					
*130-139*	26.33	1.05 (0.64-1.75)	0.28 (0.17-0.46)	0.22 (0.14-0.37)	0.05 (0.03-0.09)
*140-149*	41.62	2.54 (1.94-3.32)	1.06 (0.81-1.38)	0.86 (0.65-1.12)	0.20 (0.15-0.26)
**USA**					
Overall	12.46	2.98 (2.62-3.40)	0.37 (0.33-0.42)	0.30 (0.26-0.34)	0.07 (0.06-0.08)
Age group in years					
*75-79*	2.00	3.55 (2.66-4.72)	0.07 (0.05-0.09)	0.06 (0.04-0.08)	0.01 (0.01-0.02)
*≥80*	3.19	6.91 (5.78-8.27)	0.22 (0.18-0.26)	0.18 (0.15-0.21)	0.04 (0.04-0.05)
Gender					
*Male*	6.06	3.29 (2.78-3.89)	0.20 (0.17-0.24)	0.16 (0.14-0.19)	0.04 (0.03-0.04)
*Female*	6.40	2.62 (2.13-3.22)	0.17 (0.14-0.21)	0.14 (0.11-0.17)	0.03 (0.03-0.04)
SBP, mm Hg					
*130-139*	4.49	2.74 (2.19-3.45)	0.12 (0.10-0.15)	0.10 (0.08-0.13)	0.02 (0.02-0.03)
*140-149*	3.75	2.76 (2.14-3.54)	0.10 (0.08-0.13)	0.08 (0.07-0.11)	0.02 (0.02-0.03)
*≥150*	4.22	3.41 (2.78-4.18)	0.14 (0.12-0.18)	0.12 (0.10-0.14)	0.03 (0.02-0.03)

SBP – systolic blood pressure, BP – blood pressure

*Values presented as millions (95% CI) unless otherwise specified.

## DISCUSSION

We found that a substantial percentage of Chinese and US elderly met the eligibility criteria for intensive BP treatment; an estimated 85.39 million and 12.46 million elderly met the STEP and SPRINT eligibility criteria, representing 38.89% in China and 23.77% in the US among the ≥60 years age group, respectively. Additionally, we found that 5.78 million US (46.36%) meeting the SPRINT eligibility criteria were untreated, while the corresponding figure was 40.90 million (47.90%) in CHINA. Furthermore, we projected that 70 000 deaths in the US and 310 000 in China could be prevented or postponed if intensive BP treatment strategy was fully implemented in the eligible population.

Evidence has shown that pharmacological BP reduction provides vascular protection and would remain effective in old age [23,24]. Recently, the STEP trial in an older Chinese population demonstrated that intensive BP lowering reduced CVD events, which confirmed the benefits seen in the earlier SPRINT trial [6,7]. In our recent study, we pooled individual data from six clinical trials involving elderly hypertensive patients and proved that intensive BP treatment decreased the risk of MACE compared to standard BP treatment [8], which is in line with previous meta-analyses [9-11]. Moreover, we also estimated that the benefit became apparent after 1 year, which means many older adults would benefit from this treatment given the rising life expectancy among them [25].

Most current international or regional hypertension guidelines recommend a high BP threshold (e.g. 150/90 mm Hg) for the elderly (age ≥60 years) [16,26-28]. However, the hypertension control rate is unacceptable low worldwide, including in China [1,29], and may not be improved given the increasing ageing trend. Therefore, researchers suggested that older adults should be included in intensive BP treatment following two confirmatory SPRINT and STEP trials [12,13]; however, such revisions of guidelines would necessitate extra resources. In this analysis, we address this gap in data and found a significant increase in the percentage and numbers of elderly meeting the criteria of SPRINT and STEP in the US and China. Meanwhile, we also found that 0.31 million deaths in China and 0.07 million deaths in the US could be prevented by intensive BP treatment, and that 0.12 and 0.68 million of hypotension cases would be identified in the US and China, respectively. However, the implementation of and adherence to a more intensive BP target in practice is challenging and requires additional doctor visits and closer monitoring to avoid adverse effects.

Although our study expanded the previous analysis by including the Chinese STEP study and focusing on the elderly population in the US and China [30-32], it still has some limitations. First, the trial population did not include institutionalised participants, such as older subjects with extreme frailty or nursing home residents, so the generalisability of our findings to elderly outside the STEP- and SPRINT-eligible populations is limited. Meanwhile, with the advanced ageing trends in both countries, our estimates based on representative, but 10-year-old surveys may be underestimated. Second, NHANES did not have information on the presence of subclinical CVD, reduced left ventricular ejection, or history of medication non-adherence, which were components of the SPRINT eligibility criteria. Also, CHARLS did not have information on the STEP eligibility criteria, including coronary revascularisation within the last 12 months or severe/non-severe valvular disease. Third, although SPRINT and STEP included high CVD-risk elderly, there were still certain differences in the two trials’ inclusion and exclusion criteria. Therefore, we further estimated the numbers and percentage of SPRINT-eligible elderly in China and STEP-eligible elderly in the US. Fourthly, many side effects, such as hypotension, syncope, bradycardia, and dizziness, were defined as serious adverse events in the STEP and SPRINT trials. Of all the expected side-effects in the STEP trial, only the incidence of hypotension was significantly different between the two trial groups, which is the reason why we selected it as a side-effect in this study. Implementing a more intensive BP target in the practice should also be done in consideration of other possible side effects. Lastly, we did not have the original data for SPRINT and STEP, so we conducted a meta-analysis to calculate the average effect of intensive BP treatment on all-cause mortality risk, which may be different from the original results.

## CONCLUSIONS

We found that a substantial percentage of Chinese and US elderly met the eligibility criteria for STEP and SPRINT. The adoption of intensive BP treatment would prevent 0.31 million and 0.07 million deaths in China and the US. Considering older patients are often exposed to polypharmacy and have a higher burden of comorbidities [33], possible serious adverse events should be thoroughly investigated before the intensive BP therapy strategy is adopted, following its cost-effectiveness evaluation in both countries.

## Additional material


Online Supplementary Document
