# Supplementary material for: Generalisability and potential deaths averted from intensive blood pressure treatment among the elderly population in the US and China: A nationally representative cross-sectional study
Source: J Glob Health. 2023 Sep 8;13:04100. doi: 10.7189/jogh.13.04100 (PMC10486303; doi:10.7189/jogh.13.04100)
Supplement: Online Supplementary Document [file jogh-13-04100-s001.pdf]

# Generalizability and potential deaths averted from intensive blood pressure treatment among the elderly population in the US and China: nationally representative cross-sectional study

Online Supplementary Document

Table S1 Inclusion and exclusion criteria

Table S2 Characteristics of China elderly meeting the STEP and U.S. elderly meeting the SPRINT

Table S3 Percentage of China elderly meeting each sequential STEP eligibility criterion

Table S4 Percentage of U.S. elderly meeting each sequential SPRINT eligibility criterion

Table S5 Potential deaths averted from intensive blood pressure treatment among elderly population in the China and US according to treatment status

Figure S1 Number of Chinese adults meeting SPRINT eligibility criteria and U.S. adults meeting STEP eligibility criteria. STEP: Strategy of Blood Pressure Intervention in the Elderly Hypertensive Patients; SPRINT: Systolic Blood Pressure Intervention Trial.

Figure S2 Summary statistics and corresponding forest plot for effect of intensive blood pressure treatment on all-cause mortality risk.

Figure S3 Potential hypotension caused by intensive blood pressure treatment among elderly population in the China and US.

**Table S1.** Inclusion and exclusion criteria

| STEP | SPRINT |
|---|---|
| **Inclusion Criteria** | |
| 1. Age of 60−80 years, Han ethnicity.<br>2. SBP between 140−190 mm Hg in the three screening visits or currently under anti-hypertension treatment.<br>3. Signed the written informed consent. | 1. Age ≥ 50 years old.<br>2. SBP:<br>a) 130-180 mm Hg on 0 or 1 medication.<br>b) 130-170 mm Hg on up to 2 medications.<br>c) 130-160 mm Hg on up to 3 medications.<br>d) 130-150 mm Hg on up to 4 medications.<br>3. Risk (one or more of the following):<br>a) Presence of clinical or subclinical cardiovascular disease other than stroke.<br>b) CKD, defined as eGFR 20-59 ml/min/1.73m$^2$ within the past 6 months.<br>c) Framingham Risk Score for 10-year CVD risk≥15%.<br>d) Age ≥ 75 years. |
| **Exclusion Criteria** | |
| 1. SBP ≥ 190 mm Hg, or diastolic blood pressure (DBP) < 60 mm Hg.<br>2. Diagnosed secondary hypertension.<br>3. History of large atherosclerotic cerebral infarction or hemorrhagic stroke.<br>4. Hospitalization for myocardial infarction (MI) within the last 6 months.<br>5. Coronary revascularization within the last 12 months.<br>6. Planned to perform PCI or CABG in the next 12 months.<br>7. History of sustained atrial fibrillation or ventricular arrhythmias at entry influencing the measurement of electronic blood pressure.<br>8. New York Heart Association (NYHA) class III-IV heart failure or hospitalization for exacerbation of chronic heart failure at entry.<br>9. Severe valvular disease or valvular disease likely to require surgery or percutaneous valve replacement during the trial.<br>10. Hypertrophic cardiomyopathy (HCM).<br>11. Dilated cardiomyopathy, rheumatic heart disease, or congenital heart disease.<br>12. Uncontrolled diabetes mellitus: | 1. An indication for a specific BP lowering medication that the person is not taking.<br>2. Diagnosed secondary hypertension.<br>3. One minute standing SBP < 110 mm Hg.<br>4. Arm circumference too large or small to allow accurate blood pressure measurement with available devices.<br>5. History of stroke.<br>6. Cardiovascular event or procedure or hospitalization for unstable angina within last 3 months.<br>7. Symptomatic heart failure within the past 6 months or left ventricular ejection fraction< 35%.<br>8. Diabetes mellitus:<br>a) Taking medications for diabetes at any time in the last 12 months.<br>b) FPG at or above 126 mg/dL, A1C ≥6.5 percent, a two-hour value in an OGTT (2-h PG) at or above 200 mg/dL or a random plasma glucose concentration ≥200 mg/dL.<br>9. Proteinuria in the following ranges (within the past 6 months):<br>a) 24 hour urinary protein excretion ≥ 1 g/day, or<br>b) If measurement a) is not available, then 24 hour urinary albumin excretion ≥ 600 mg/day, or<br>c) If measurements a) or b) are not available, then spot urine protein/creatinine ratio ≥ 1 g/g creatinine, or |

| | |
|---|---|
| serum fasting glucose ≥ 200 mg/dl [11.1mmol/L], glycated hemoglobin [HbA1] > 8%).<br>13. Severe liver or kidney dysfunction: Alanine aminotransferase [ALT] ≥ 3 times the upper limit of normal value, or end stage renal disease on dialysis or estimated glomerular filtration rate [eGFR] < 30 mL/min/1.73m$^2$, or serum creatinine > 2.5 mg/dl [> 221 mol/L].<br>14. Severe somatic disease such as cancer.<br>15. Severe cognitive impairment or mental disorders.<br>16. Participating in other clinical trials. | d) If measurements a), b), or c) are not available, then spot urine albumin/creatinine ratio≥ 600 mg/g creatinine, or<br>e) If measurements a), b), c), or d) are not available, then urine dipstick ≥ 2+ protein.<br>10. Diagnosis of polycystic kidney disease.<br>11. Glomerulonephritis treated with or likely to be treated with immunosuppressive therapy.<br>12. eGFR < 20 ml/min /1.73m$^2$ or end-stage renal disease (ESRD).<br>13. A medical condition likely to limit survival to less than 3 years, or a cancer diagnosed and treated within the past 2 years.<br>14. Any factors judged by the clinic team to be likely to limit adherence to interventions.<br>15. Failure to obtain informed consent from participant.<br>16. Currently participating in another clinical trial (intervention study).<br>17. Living in the same household as an already randomized SPRINT participant.<br>18. Any organ transplant.<br>19. Unintentional weight loss > 10% in last 6 months.<br>20. Pregnancy, currently trying to become pregnant, or of child-bearing potential and not using birth control. |

STEP: Strategy of Blood Pressure Intervention in the Elderly Hypertensive Patients; SPRINT: Systolic Blood Pressure Intervention Trial; eGFR: estimated glomerular filtration rate; SBP: systolic blood pressure; CKD: chronic kidney disease; CVD: cardiovascular disease; DBP: diastolic blood pressure; BP: blood pressure; PCI: percutaneous coronary intervention; CABG: coronary artery bypass grafting; OGTT: oral glucose tolerance test.

**Table S2.** Characteristics of China elderly meeting the STEP and U.S. elderly meeting the SPRINT

| | | China | U.S. |
|---|---|---|---|
| Sample, N | | 85.39 | 12.46 |
| Age group, yrs | | 68.02±5.49 | 71.81±6.98 |
| | 60-74 | 71.28 (83.48%) | 7.27 (58.35%) |
| | 75-79 | 13.77 (16.13%) | 2.00 (16.04%) |
| | ≥80 | 0.33 (0.39%) | 3.19 (25.61%) |
| Gender | | | |
| | Male | 40.64 (47.60%) | 6.06 (48.62%) |
| | Female | 44.74 (52.40%) | 6.40 (51.38%) |
| Currently smoking | | | |
| | Yes | 22.29 (26.11%) | 1.41 (11.30%) |
| | No | 63.10 (73.89%) | 11.05 (88.70) |
| Framingham risk score | | 0.32±0.18 | 0.28±0.13 |
| | <10% | 2.71 (4.71%) | 0.13 (1.15%) |
| | 10%-20% | 14.97 (26.00%) | 3.94 (34.34%) |
| | >20% | 39.90 (69.29%) | 7.40 (64.51%) |
| SBP, mm Hg | | 150.85±17.42 | 146.67±12.47 |
| | <140 | 17.44 (20.43%) | 4.49 (36.00%) |
| | 140-149 | 26.33 (30.84%) | 3.75 (30.10%) |
| | ≥150 | 41.62 (48.74%) | 4.22 (33.90%) |
| DBP, mm Hg | | 81.63±10.36 | 69.55±16.25 |
| | <80 | 37.54 (43.96%) | 9.46 (75.90%) |
| | 80-89 | 29.63 (34.70%) | 2.13 (17.10%) |
| | ≥90 | 18.23 (21.34%) | 0.87 (7.00%) |

Values are number (%) in millions or Mean ± SD.
STEP: Strategy of Blood Pressure Intervention in the Elderly Hypertensive Patients; SPRINT: Systolic Blood Pressure Intervention Trial; SBP: systolic blood pressure; DBP: diastolic blood pressure.

**Table S3.** Percentage of China elderly meeting each sequential STEP eligibility criterion*

| | Elderly Population (Millions) | +Age Criteria (Millions) | +SBP Criteria (Millions) | +No Exclusion Criteria (Millions) |
|---|---|---|---|---|
| **Overall population** | | | | |
| Overall | 219.56 | 91.13 (90-92.15) | 45.70 (43.76-47.66) | 38.89 (36.97-40.84) |
| Age group, yrs | | | | |
| 60-69 | 130.25 | 100 | 46.08 (43.59-48.59) | 40.25 (37.72-42.84) |
| 70-80 | 69.84 | 100 | 57.75 (54.18-61.24) | 47.20 (43.70-50.72) |
| Gender | | | | |
| Male | 109.87 | 93.29 (91.91-94.44) | 43.78 (40.78-46.83) | 36.99 (34.01-40.08) |
| Female | 109.69 | 88.97 (87.14-90.57) | 47.63 (45.15-50.12) | 40.79 (38.39-43.24) |
| SBP, mm Hg | | | | |
| 140-149 | 32.19 | 91.67 (89.12-93.66) | 91.67 (89.12-93.66) | 81.79 (78.39-84.75) |
| ≥150 | 58.78 | 85.49 (82.54-88.02) | 82.25 (79.14-84.98) | 70.80 (66.91-74.41) |
| **Treated hypertension** | | | | |
| Overall | 61.01 | 90.38 (87.84-92.43) | 90.38 (87.84-92.43) | 72.93 (69.26-76.31) |
| Age group, yrs | | | | |
| 60-69 | 34.77 | 100 | 100 | 83.02 (79.47-86.06) |
| 70-80 | 20.37 | 100 | 100 | 76.72 (69.81-82.44) |
| Gender | | | | |
| Male | 28.35 | 92.16 (88.64-94.65) | 92.16 (88.64-94.65) | 73.39 (66.98-78.95) |
| Female | 32.66 | 88.83 (85.03-91.76) | 88.83 (85.03-91.76) | 72.52 (68.33-76.35) |
| SBP, mm Hg | | | | |
| 140-149 | 11.25 | 94.08 (89.58-96.71) | 94.08 (89.58-96.71) | 78.37 (71.44-84.00) |
| ≥150 | 25.45 | 86.68 (81.81-90.40) | 86.68 (81.81-90.40) | 71.63 (65.54-77.03) |
| **Without treated hypertension** | | | | |
| Overall | 158.55 | 91.42 (90.14-92.55) | 28.51 (26.66-30.45) | 25.79 (24.07-27.60) |
| Age group, yrs | | | | |
| 60-69 | 95.48 | 100 | 26.44 (24.39-28.61) | 24.68 (22.66-26.81) |
| 70-80 | 49.47 | 100 | 40.35 (36.21-44.64) | 35.04 (31.24-39.04) |
| Gender | | | | |
| Male | 81.52 | 93.68 (92.17-94.91) | 26.96 (24.41-29.68) | 24.33 (21.96-26.87) |
| Female | 77.03 | 89.03 (86.88-90.87) | 30.16 (27.51-32.95) | 27.34 (24.87-29.96) |
| SBP, mm Hg | | | | |
| 140-149 | 20.94 | 90.37 (87.03-92.92) | 90.37 (87.03-92.92) | 83.62 (79.79-86.85) |
| ≥150 | 33.33 | 84.58 (80.72-87.79) | 78.86 (74.77-82.44) | 70.16 (65.05-74.82) |

Values are % (95% confidence interval) unless otherwise specified.

STEP: Strategy of Blood Pressure Intervention in the Elderly Hypertensive Patients; SBP: systolic blood pressure.

*Participants were ≥60 years of age.

**Table S4.** Percentage of U.S. elderly meeting each sequential SPRINT eligibility criterion*

| | Elderly Population (Millions) | +SBP Criteria (Millions) | +high CVD risk (Millions) | +No Exclusion Criteria (Millions) |
|---|---|---|---|---|
| **Overall population** | | | | |
| Overall | 52.41 | 49.56 (47.78-51.34) | 42.55 (40.82-44.29) | 23.77 (22.32-25.28) |
| Age group, yrs | | | | |
| 75-79 | 6.57 | 55.84 (51.58-60.00) | 55.84 (51.58-60.00) | 30.44 (26.70-34.46) |
| ≥80 | 9.42 | 61.74 (58.47-64.92) | 61.74 (58.47-64.92) | 33.86 (30.68-37.18) |
| Gender | | | | |
| Male | 23.46 | 46.52 (43.92-49.13) | 45.07 (42.49-47.67) | 25.83 (23.64-28.14) |
| Female | 28.96 | 52.02 (49.59-54.45) | 40.50 (38.19-42.85) | 22.11 (20.21-24.13) |
| SBP, mm Hg | | | | |
| 130-139 | 10.73 | 100 | 76.96 (73.06-80.45) | 41.79 (38.04-45.64) |
| 140-149 | 7.51 | 100 | 87.10 (83.11-90.25) | 49.95 (45.27-54.62) |
| ≥150 | 8.87 | 87.20 (84.69-89.35) | 84.55 (81.75-86.99) | 47.61 (43.70-51.56) |
| **Treated hypertension** | | | | |
| Overall | 27.75 | 53.41 (51.05-55.77) | 49.21 (46.86-51.57) | 24.09 (22.12-26.17) |
| Age group, yrs | | | | |
| 75-79 | 4.03 | 60.28 (54.99-65.34) | 60.28 (54.99-65.34) | 28.50 (23.85-33.65) |
| ≥80 | 5.94 | 63.50 (59.32-67.47) | 63.50 (59.32-67.47) | 32.35 (28.34-36.64) |
| Gender | | | | |
| Male | 11.74 | 49.52 (45.91-53.14) | 49.40 (45.79-53.01) | 24.18 (21.22-27.40) |
| Female | 16.01 | 56.26 (53.16-59.32) | 49.08 (45.98-52.19) | 24.02 (21.43-26.81) |
| SBP, mm Hg | | | | |
| 130-139 | 5.69 | 100 | 86.85 (82.42-90.29) | 41.45 (36.60-46.47) |
| 140-149 | 4.28 | 100 | 92.46 (87.27-95.64) | 49.27 (43.15-55.42) |
| ≥150 | 5.58 | 87.02 (83.88-89.63) | 85.33 (81.87-88.22) | 39.75 (34.93-44.78) |
| **Without treated hypertension** | | | | |
| Overall | 24.67 | 45.22 (42.56-47.92) | 35.04 (32.59-37.58) | 23.42 (21.32-25.65) |
| Age group, yrs | | | | |
| 75-79 | 2.53 | 48.76 (41.86-55.71) | 48.76 (41.86-55.71) | 33.54 (27.47-40.22) |
| ≥80 | 3.48 | 58.75 (53.41-63.9) | 58.75 (53.41-63.90) | 36.43 (31.36-41.81) |
| Gender | | | | |
| Male | 11.72 | 43.51 (39.79-47.3) | 40.73 (37.10-44.48) | 27.48 (24.32-30.88) |
| Female | 12.95 | 46.78 (43-50.60) | 29.89 (26.69-33.31) | 19.74 (17.07-22.71) |
| SBP, mm Hg | | | | |
| 130-139 | 5.04 | 100 | 65.81 (59.45-71.65) | 42.18 (36.44-48.14) |
| 140-149 | 3.23 | 100 | 79.98 (73.12-85.44) | 50.84 (43.62-58.02) |
| ≥150 | 3.30 | 87.49 (82.97-90.95) | 83.22 (78.15-87.31) | 60.91 (54.64-66.84) |

Values are % (95% confidence interval) unless otherwise specified.
SPRINT: Systolic Blood Pressure Intervention Trial; CVD: cardiovascular disease; SBP: systolic blood pressure.
*****Participants were ≥60 years of age.

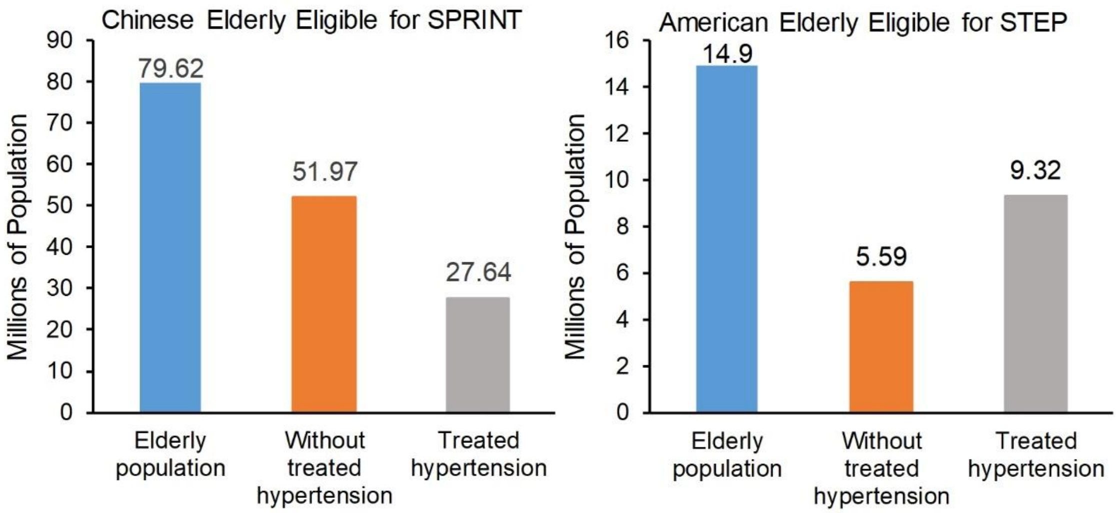


**Figure S1.** Number of Chinese adults meeting SPRINT eligibility criteria and U.S. adults meeting STEP eligibility criteria. STEP: Strategy of Blood Pressure Intervention in the Elderly Hypertensive Patients; SPRINT: Systolic Blood Pressure Intervention Trial.

**Table S5.** Potential deaths averted from intensive blood pressure treatment among elderly population in the China and US according to treatment status

| | Elderly Population* | Death rate per 100 person years | Standard group* | Intensive BP group* | Difference* |
|---|---|---|---|---|---|
| **China** | | | | | |
| **Treated hypertension** | | | | | |
| Overall | 44.49 | 2.13 (1.60-2.83) | 0.95 (0.71-1.26) | 0.77 (0.58-1.02) | 0.18 (0.14-0.24) |
| Age group, yrs | | | | | |
| 60-69 | 28.86 | 1.40 (0.91-2.15) | 0.40 (0.26-0.62) | 0.33 (0.21-0.50) | 0.08 (0.05-0.12) |
| 70-80 | 15.63 | 3.67 (2.50-5.39) | 0.57 (0.39-0.84) | 0.46 (0.32-0.68) | 0.11 (0.07-0.16) |
| Gender | | | | | |
| Male | 20.81 | 2.86 (1.97-4.14) | 0.60 (0.41-0.86) | 0.48 (0.33-0.70) | 0.11 (0.08-0.16) |
| Female | 23.68 | 1.55 (0.99-2.42) | 0.37 (0.23-0.57) | 0.30 (0.19-0.46) | 0.07 (0.04-0.11) |
| SBP, mm Hg | | | | | |
| 140-149 | 8.82 | 2.11 (1.10-4.05) | 0.19 (0.10-0.36) | 0.15 (0.08-0.29) | 0.04 (0.02-0.07) |
| ≥150 | 18.23 | 2.41 (1.57-3.69) | 0.44 (0.29-0.67) | 0.36 (0.23-0.54) | 0.08 (0.05-0.13) |
| **Without treated hypertension** | | | | | |
| Overall | 40.90 | 1.72 (1.25-2.36) | 0.70 (0.51-0.97) | 0.57 (0.41-0.78) | 0.13 (0.10-0.18) |
| Age group, yrs | | | | | |
| 60-69 | 23.56 | 1.10 (0.66-1.83) | 0.26 (0.16-0.43) | 0.21 (0.13-0.35) | 0.05 (0.03-0.08) |
| 70-80 | 17.33 | 2.70 (1.80-4.07) | 0.47 (0.31-0.70) | 0.38 (0.25-0.57) | 0.09 (0.06-0.13) |
| Gender | | | | | |
| Male | 19.83 | 2.06 (1.36-3.13) | 0.41 (0.27-0.62) | 0.33 (0.22-0.50) | 0.08 (0.05-0.12) |
| Female | 21.06 | 1.40 (0.86-2.28) | 0.29 (0.18-0.48) | 0.24 (0.15-0.39) | 0.06 (0.03-0.09) |
| SBP, mm Hg | | | | | |
| 140-149 | 17.51 | 0.60 (0.27-1.34) | 0.11 (0.05-0.23) | 0.09 (0.04-0.19) | 0.02 (0.01-0.04) |
| ≥150 | 23.38 | 2.63 (1.86-3.73) | 0.62 (0.44-0.87) | 0.50 (0.35-0.71) | 0.12 (0.08-0.17) |
| **U.S.** | | | | | |
| **Treated hypertension** | | | | | |
| Overall | 6.68 | 2.80 (2.33-3.38) | 0.19 (0.16-0.23) | 0.15 (0.13-0.18) | 0.04 (0.03-0.04) |
| Age group, yrs | | | | | |
| 75-79 | 1.15 | 2.53 (1.61-3.97) | 0.03 (0.02-0.05) | 0.02 (0.02-0.04) | 0.01 (0.00-0.01) |
| ≥80 | 1.92 | 6.47 (5.05-8.28) | 0.12 (0.10-0.16) | 0.10 (0.08-0.13) | 0.02 (0.02-0.03) |
| Gender | | | | | |
| Male | 2.84 | 3.05 (2.36-3.94) | 0.09 (0.07-0.11) | 0.07 (0.05-0.09) | 0.02 (0.01-0.02) |
| Female | 3.85 | 2.58 (1.97-3.37) | 0.10 (0.08-0.13) | 0.08 (0.06-0.11) | 0.02 (0.01-0.02) |
| SBP, mm Hg | | | | | |
| 130-139 | 2.36 | 2.58 (1.86-3.57) | 0.06 (0.04-0.08) | 0.05 (0.04-0.07) | 0.01 (0.01-0.02) |
| 140-149 | 2.11 | 2.44 (1.70-3.51) | 0.05 (0.04-0.07) | 0.04 (0.03-0.06) | 0.01 (0.01-0.01) |
| ≥150 | 2.22 | 3.35 (2.51-4.48) | 0.07 (0.06-0.10) | 0.06 (0.05-0.08) | 0.01 (0.01-0.02) |
| **Without treated hypertension** | | | | | |
| Overall | 5.78 | 3.18 (2.65-3.81) | 0.18 (0.15-0.22) | 0.15 (0.12-0.18) | 0.03 (0.03-0.04) |
| Age group, yrs | | | | | |
| 75-79 | 0.85 | 4.88 (3.37-7.06) | 0.04 (0.03-0.06) | 0.03 (0.02-0.05) | 0.01 (0.01-0.01) |
| ≥80 | 1.27 | 7.48 (5.77-9.70) | 0.09 (0.07-0.12) | 0.08 (0.06-0.10) | 0.02 (0.01-0.02) |
| Gender | | | | | |
| Male | 3.22 | 3.50 (2.80-4.37) | 0.11 (0.09-0.14) | 0.09 (0.07-0.11) | 0.02 (0.02-0.03) |

| | | | | | |
|---|---|---|---|---|---|
| Female | 2.56 | 2.68 (1.95-3.68) | 0.07 (0.05-0.09) | 0.06 (0.04-0.08) | 0.01 (0.01-0.02) |
| SBP, mm Hg | | | | | |
| 130-139 | 2.13 | 2.93 (2.13-4.02) | 0.06 (0.05-0.09) | 0.05 (0.04-0.07) | 0.01 (0.01-0.02) |
| 140-149 | 1.64 | 3.12 (2.21-4.41) | 0.05 (0.04-0.07) | 0.04 (0.03-0.06) | 0.01 (0.01-0.01) |
| ≥150 | 2.01 | 3.47 (2.60-4.63) | 0.07 (0.05-0.09) | 0.06 (0.04-0.08) | 0.01 (0.01-0.02) |

*Values are number (95% confidence interval) in millions.
SBP: systolic blood pressure; BP: blood pressure.

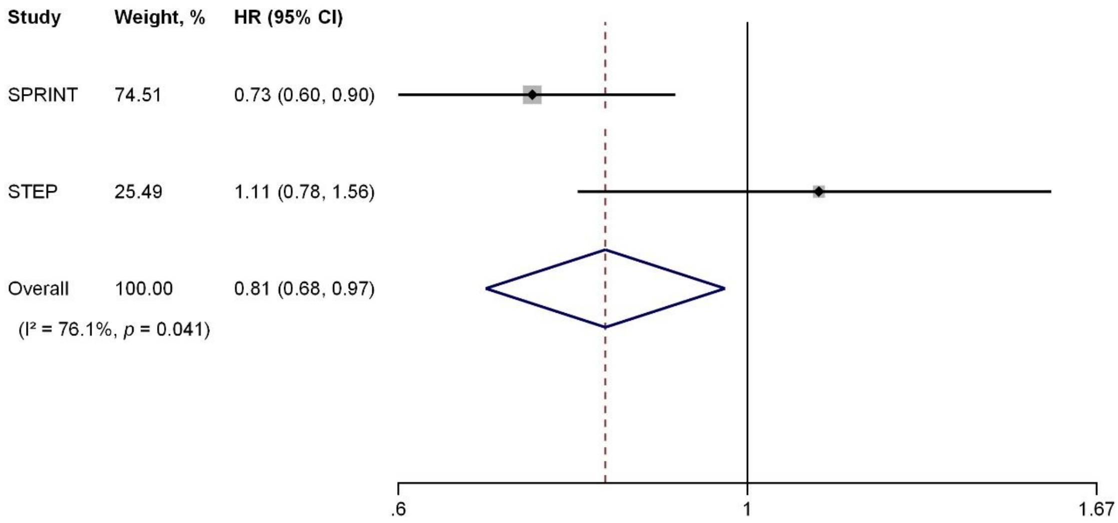


**Figure S2.** Summary statistics and corresponding forest plot for effect of intensive blood pressure treatment on all-cause mortality risk.

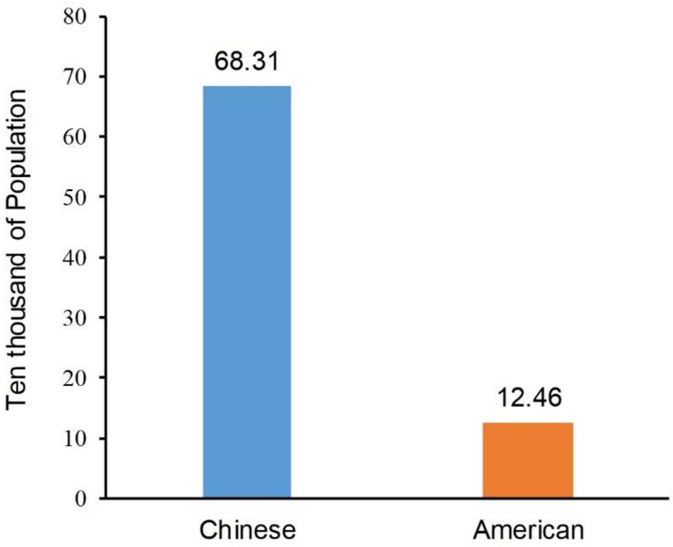


**Figure S3.** Potential hypotension caused by intensive blood pressure treatment among elderly population in the China and US.
